# Ultra-Stretchable and Self-Healing Anti-Freezing Strain Sensors Based on Hydrophobic Associated Polyacrylic Acid Hydrogels

**DOI:** 10.3390/ma14206165

**Published:** 2021-10-18

**Authors:** Shuya Yin, Gehong Su, Jiajun Chen, Xiaoyan Peng, Tao Zhou

**Affiliations:** 1 State Key Laboratory of Polymer Materials Engineering of China, Polymer Research Institute, Sichuan University, Chengdu 610065, China; yinshuya@163.com (S.Y.); 18782940146@163.com (G.S.); 2019223090023@stu.scu.edu.cn (J.C.); 2019223090148@stu.scu.edu.cn (X.P.); 2 College of Science, Sichuan Agricultural University, Ya’an 625014, China

**Keywords:** antifreeze hydrogels, hydrophobic associated polyacrylic acid, ultra-stretchable, self-healing, sensors

## Abstract

Water-rich conductive hydrogels with excellent stretchability are promising in strain sensors due to their potential application in flexible electronics. However, the features of being water-rich also limit their working environment. Hydrogels must be frozen at subzero temperatures and dried out under ambient conditions, leading to a loss of mechanical and electric properties. Herein, we prepare HAG_x_ hydrogels (a polyacrylic acid (HAPAA) hydrogel in a binary water–glycerol solution, where x is the mass ratio of water to glycerol), in which the water is replaced with water–glycerol mixed solutions. The as-prepared HAG_x_ hydrogels show great anti-freezing properties at a range of −70 to 25 °C, as well as excellent moisture stability (the weight retention rate was as high as 93% after 14 days). With the increase of glycerol, HAG_x_ hydrogels demonstrate a superior stretchable and self-healing ability, which could even be stretched to more than 6000% without breaking, and had a 100% self-healing efficiency. The HAG_x_ hydrogels had good self-healing ability at subzero temperatures. In addition, HAG_x_ hydrogels also had eye-catching adhesive properties and transparency, which is helpful when used as strain sensors.

## 1. Introduction

Hydrogels are a promising and versatile material. Water-rich properties and cross-linked polymer networks endow the materials with liquid-like transport properties and solid-like mechanical properties, respectively [1,2]. Based on these intrinsic traits, hydrogels have developed a lot of applications in different fields, such as in flexible electronics [3], drug delivery [4], tissue engineering [5], waste treatment [6,7], and superabsorbent materials [8]. Due to the outstanding performance in artificial nerves [9], flexible robotics [10], solid electrolytes [11,12,13,14], conductors [15], tactile sensors [16], environment sensors [17,18], human–machine interface [9,19], optoelectronics [20,21], and energy applications [22,23,24,25], flexible electronics have earned more extensive and particular attention during practical utilization [26].

However, most water-rich hydrogels used for electronic sensors can freeze, turning hard and fragile at subzero temperatures, which restricts the transport of conductive ions and makes them lose their excellent original properties [27,28]. Not coincidentally, the hydrogels cannot avoid dehydration and quickly dry out under ambient conditions [29]. Hence, such hydrogels cannot be used directly and usually need to be encapsulated to prevent water evaporation. Subzero temperature and dehydration intolerance remain big challenges and seriously impede their development.

Fortunately, with the efforts of many researchers, two main strategies have gradually been developed to achieve the anti-freezing property of hydrogels. One is dependent on the colligative properties of a mixed solution [30], which is made by mixing high concentrations of salts with the aqueous solution to depress the freezing point. For example, calcium chloride (CaCl_2_) and lithium chloride (LiCl) were extensively used to prevent ice formation [31,32]. Vlassak and co-workers [33] prepared a polyacrylamide (PAAm) hydrogel with the freezing point at −57 °C through the addition of CaCl_2_. The other strategy is to replace the water-rich hydrogels with organohydrogels [32,34,35] by changing the traditional water phase with a binary mixed solution of water and organic solvents, such as ethylene glycol (EG) and glycerol water solution. With the addition of glycerol/H_2_O organic solvent, Lu prepared the PAAm-PAA hydrogels, which worked well even at a temperature of −20 °C [36]. Similarly, Li designed a multifunctional glycerol/H_2_O PAAm hydrogel with a lowest transition temperature of −25.59 °C [37]. Liu also reported that when the mass ratio of EG to H_2_O was 2:1, the PVA hydrogel could still maintain flexibility and strain sensitivity even when the temperature was as low as −40 °C [38]. Not only can the organohydrogels endow the hydrogels with anti-freezing properties, they also avoid the severe water dehydration problem under ambient conditions. In addition, good self-healing ability is important for the hydrogels’ life extension and maintenance [39,40]. However, to the best of our knowledge, the self-healing ability of many anti-freezing hydrogels reported is relatively poor. Thus, for anti-freezing hydrogels, a better self-healing capability is necessary for practical applications.

Herein, based on the existing research and unsolved problems, we found that by replacing the water with a binary water–glycerol solution in the hydrophobic associated polyacrylic acid (HAPAA) hydrogel, a conductive, anti-freezing, self-healing, stretchable, and adhesive hydrogel (named HAG_x_ hydrogel, where x is the mass ratio of water to glycerol) could be fabricated. The polarity of the water–glycerol solution is lower than that of water, which has a significant influence on the intensity of hydrophobic association. In addition, the addition of glycerol introduced more hydrogen bonding interactions in HAG_x_ hydrogels. Differently from the single dynamic crosslinking [41], a hierarchical system of supramolecular association (hydrophobic association and hydrogen bonds in HAG_x_ hydrogels) usually provides excellent stretchability and self-healing ability [42,43,44]. In this work, with the increase of glycerol, HAG_x_ hydrogels show superior stretchable and self-healing ability, and could be stretched to more than 6000% without breaking and had a 100% healing efficiency. In addition, the as-prepared HAG_x_ hydrogels also demonstrated great anti-freezing properties at a range of −70 to 25 °C, as well as moisture stability (the weight retention rate was as high as 93% after 14 days). Although no additional conductive filler was added to HAG_x_ hydrogels, the existence of dodecyltrimethylammonium bromide (CTAB) cationic micelles and initiator ions (APS) endowed the HAG_x_ hydrogels with electrical conductivity.

## 2. Experimental Section

### 2.1. Materials

Acrylic acid (AA, liquid, 99.9%), lauryl methacrylate (LMA, liquid, 99.6%), hexadecyl trimethyl ammonium bromide (CTAB, powder, 99.9%), and glycerol were purchased from Adamas-beta Co. Ltd. (Shanghai, China) Ammonium persulfate (APS), with analytical grade provided by Kelong Chemical Reagent Company (Chengdu, China). All reagents were used as received without any further purification.

### 2.2. Synthesis of HAG_x_ Hydrogels

Typically, the hydrogels were prepared via three steps. Firstly, glycerol was mixed with water and quickly stirred for 15 min; the mass ratio of glycerol to water can be seen in Table S1. Secondly, LMA and CTAB were added to the water–glycerol mixed solution. After continuously stirring for 4 h, AA monomer was added to the above solution. Finally, the appropriate amount of APS was dissolved to initiate the polymerization. The mixed solution was deoxygenized and then poured into two sealed glass plates to react for 6 h at 60 °C. After that, the final HAG_x_ hydrogels were obtained. Table S2 shows the detailed compositions of HAPAA and HAG_x_ hydrogels.

### 2.3. Characterizations

Nicolet iS50 Fourier transform infrared spectrometer was used to record the ATR FTIR spectra of HAPAA and HAG_x_ hydrogels. For each spectrum, 20 scans were conducted with a spectral resolution of 4 cm^−1^ in the region from 4000 cm^−1^ to 650 cm^−1^.

Rheological experiments of the HAG_11_ hydrogel were performed on the AR 2000EX rheometer (TA instrument) using a parallel plate (25 mm in diameter). The hydrogel was prepared as round shaped disks with a thickness of 1 mm, which was tested at a fixed strain amplitude of 1%, using a frequency sweep mode from 0.1 to 100 rad·s^−1^, and the tested temperature was 25 °C.

Differential scanning calorimeter (DSC) measurements were performed for detecting the freezing temperature of HAG_x_ hydrogels using NETZSCH DSC 204 F1. The sample weight was 8 mg. The cooling process was taken from 25 °C to −70 °C with a cooling rate of 5 °C·min^−1^. After 10 min of balancing, it was heated up to 25 °C at a rate of 5 °C·min^−1^.

The anti-freezing property of hydrogels was carefully inspected according to the subzero freezing behavior at temperatures of −20 °C and −70 °C. Under an extreme circumstance, the state of the hydrogels was displayed by close observation or ascertained by their deformation under external force. In addition, in order to test the properties at low temperature and get the stress–strain curves of the HAG_x_ hydrogels, they were frozen at −20 °C for 24 h and taken out to perform the test immediately.

The moisture stability of HAPAA and HAG_x_ hydrogels was assessed through the weight loss of the hydrogels at 25 °C and a humidity of 60% for 15 days. During the dehydration process, the weight of HAPAA and HAG_x_ hydrogels was recorded at a fixed time.

To evaluate the self-healing ability of HAG hydrogels, first, a dumbbell-shaped hydrogel sample was completely cut into two halves. Then, these two halves were connected with each other and placed at ambient conditions (25 °C, relative humidity 50%) for 24 h to allow them to self-heal. The self-healing efficiency of HAG gels was evaluated using the recovery of the elongation at break (defined as (ε_s_/ε_0_) × 100%, where ε_s_ and ε_0_ are the tensile strain of the healed and original sample, respectively).

All the mechanical tests were performed on a versatile testing machine (Instron 5966, Instron Corporation, New York, NY, USA) with a 1 kN load cell. For the tensile tests, the samples were tailored to rectangles (2 mm in thickness, 5 mm in width, and 30 mm in length), and the stretching speed was fixed at 100 mm·min^−1^. The self-healing efficiency (η) of the hydrogels was defined as η = λ_max_/λ_0_ × 100%, where λ_max_ and λ_0_ correspond to the max strain before and after healing, respectively. The characterization of the adhesive properties with different substrates was also taken by the versatile testing machine. Briefly, using stainless steel as an example, the HAG_11_ hydrogel was sandwiched between two stainless steel plates with a bonding area of 15 mm × 15 mm. Then the sample was pressed with a piece of cast iron for 12 h before the adhesion tests, and the stretching speed was 100 mm·min^−1^. The adhesive strength was calculated by the maximum force divided by the bonding area.

An ultraviolet spectrophotometer (UV-2600, Shimadzu Corporation, Tokyo, Japan) was used to confirm the transparency of the HAPAA and HAG_11_ hydrogels. The spectra were collected in the region of 800–400 nm with a resolution of 0.1 nm.

The digital multimeter (UT181A, UNIT Corporation, Dongguan, China) was used to monitor and record the resistance signals of the strain sensors dynamically.

## 3. Results and Discussion

**Figure 1:**
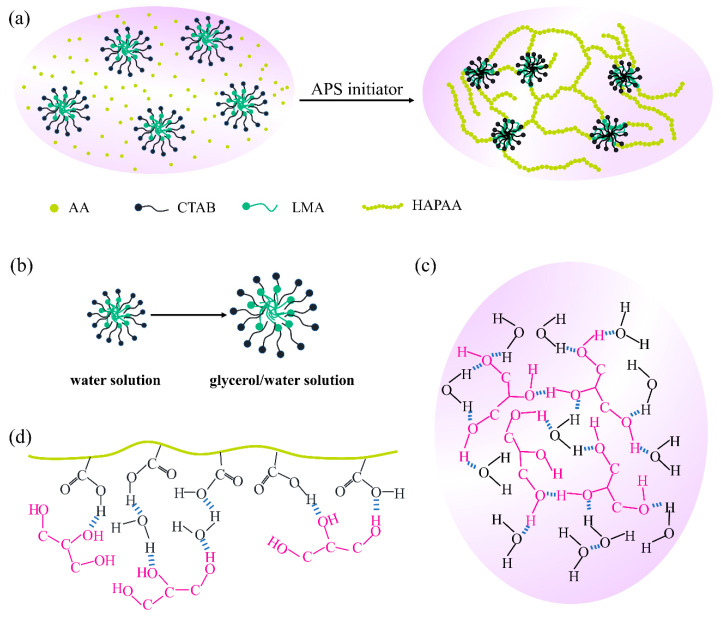
(**a**) Polymerization and the structure of HAG_x_ hydrogels. (**b**) The association of the micelles in water and glycerol/water solution. (**c**) Hydrogen bonds between water and glycerol. (**d**) Hydrogen bonds between HAPAA and glycerol.

The synthesis method of HAG_x_ hydrogels is maneuverable. The hydrophobic monomer LMA and surfactant CTAB are dissolved in water–glycerol mixed solutions to form micelles, then the monomer AA is added. After mixing evenly, APS is added to initiate the polymerization reaction (Figure 1a). The association strength of micelles is greatly affected by the polarity of the mixed solution [45,46,47,48]. In general, in a weakly polar solvent, the micellar association is loose. As the polarity of glycerol is lower than that of water, the polarity of the water–glycerol mixed solution is lower than that of the neat water, and as the mass ratio of glycerol increases, the polarity of the mixed solution will become weaker and weaker. Figure 1b is the schematic of the association state of micelles under different polarities. It is known that, between glycerol and water, strong hydrogen bonding interactions can be formed (Figure 1c), which not only weakens the evaporation of water but also prevents the formation of crystal ice [32,33,48]. In addition, the binary water–glycerol organic solvents can also form non-covalent interactions with HAPAA molecular chains by hydrogen bonds (Figure 1d). A large number of hydrogen bonds have a great influence on the performance of the HAG_x_ hydrogels.

The ATR-FTIR spectra in Figure S1 (Supporting Information) were collected to study the structure of HAG_x_ hydrogels and the interaction between glycerol and the HAPAA hydrogel network. It is found that the −OH stretching vibration of HAG_11_ (3381 cm^−1^) is between HAPAA (3396 cm^−1^) and glycerol (3319 cm^−1^), which proves that the −OH of glycerol has an interaction with −COOH of HAPAA. In addition, the C−O stretching vibration of glycerol in HAG_11_ moves to a higher wavenumber compared with the pure glycerol. Based on the above discussion, it is reasonable to believe that glycerol has an interaction with HAPAA molecular chains. The density functional theory (DFT) calculations were carried out to explore the role of glycerol in HAG_x_ hydrogels (Figure S2, Table S3). The analysis showed that the interaction of water–glycerol is stronger than that of water–water and glycerol–glycerol. Besides, the interaction of water–glycerol with HAPAA is also stronger than water–HAPAA and glycerol–HAPAA. Rheological measurement was used to study the crosslinking network of HAG hydrogels (Figure S3) [49]. It can be seen that the storage modulus (G′) of the hydrogels is higher than the loss modulus (G″). With the increase of glycerol, the loss modulus gradually increases. These results indicate that the addition of glycerol reduces the elasticity of HAG_x_ hydrogels and increases the viscous flow.

### 3.1. Anti-Freezing Property and Moisture Stability

Different from the water–based hydrogels, the binary water–glycerol-based HAG_x_ hydrogels exhibit eye-catching subzero temperature tolerance and moisture stability. As a well-known anti-freezing agent, the phase diagram (Figure S4) shows the relationship between the freezing points and the mass ratio of the glycerol–water mixed solutions. From the phase diagram, it is learned that when the mass ratio of glycerol is 50–80%, the freezing points of the mixed solution very easily fall below −20 °C. For HAG_x_ hydrogels, the freezing point is certainly lower with the help of water and glycerol.

**Figure 2:**
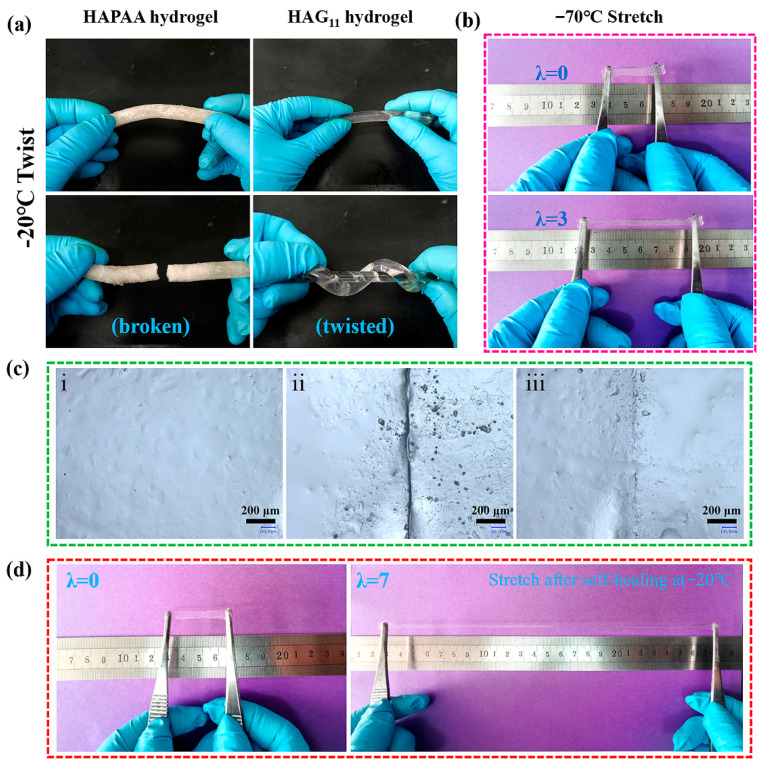
(**a**) Photos of the twisted HAPAA and HAG_11_ hydrogels at −20 °C. (**b**) The original and stretched HAG_11_ hydrogel at −70 °C. (**c**) Microscope images of HAG_11_ hydrogels: (i) original, (ii) cutting off and being contacted immediately, and (iii) self-healing for 3 days at −20 °C. (**d**) Photos of the original and the self-healed HAG_11_ hydrogels for 3 days at −20 °C.

**Figure 3:**
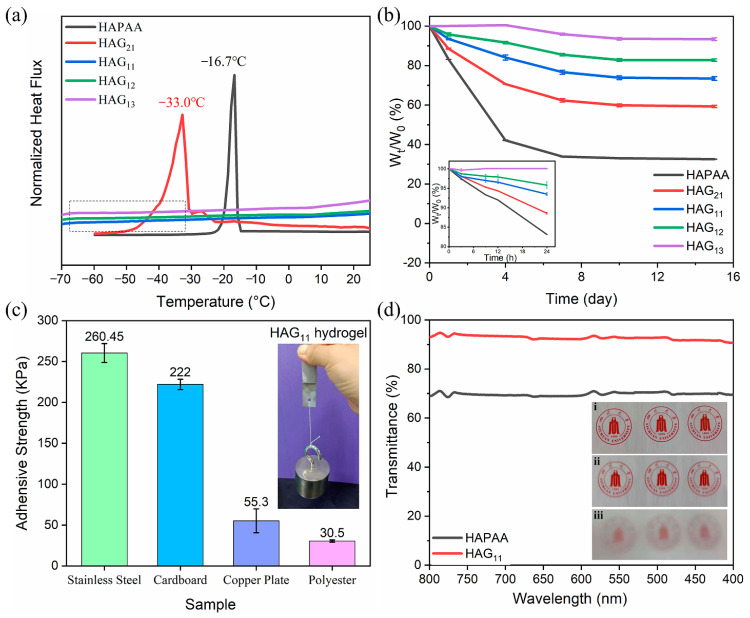
(**a**) DSC curves of HAPAA and HAG_x_ hydrogels. (**b**) The water-retention of HAPAA and HAG_x_ hydrogels with a testing time lasting for 15 days. (**c**) The adhesive strength of the HAG_11_ hydrogel to different sample plates. The inset image shows the bonded plates of stainless steel lifted a weight of 1 kg. (**d**) The transmittance of HAPAA and HAG_11_ hydrogels with a thickness of 2 mm. The inset images are (i) a real photo directly taken by a mobile phone, (ii) a photo taken by a mobile phone with the phone lens covered by HAG_11_ hydrogel, and (iii) a photo taken by a mobile phone with the phone lens covered by HAPAA hydrogel.

To specifically demonstrate the anti-freezing property, the HAPAA and HAG_11_ hydrogels were frozen at −20 °C for 24 h (Figure 2a). HAPAA hydrogels are entirely frozen and can be broken easily, while the HAG_11_ hydrogel maintains strong mechanical properties and can even be twisted at −20 °C. The thermal properties were conducted using DSC from −70 °C to 25 °C (Figure 3a). For the HAPAA hydrogel, a crystalline peak at −16.7 °C is clearly observed. When a small amount of glycerol was added (for the sample of HAG_21_), the freezing point dropped to −33.0 °C. Note that, as the content of glycerol increases further (for samples of HAG_11_, HAG_12_, and HAG_13_), the DSC curves are entirely flat, showing no peaks from −70 °C to 25 °C, which indicates that the obtained HAG_x_ hydrogels have excellent anti-freezing properties. Based on DSC curves, to specifically show the anti-freezing properties, we stored the HAG_11_ hydrogel at −70 °C for six hours, which was then taken out and stretched immediately. As shown in Figure 2b, the HAG_11_ hydrogel can still be stretched to about three times the original length. This phenomenon clearly indicates that the HAG_11_ hydrogel has an excellent anti-freezing properties and can even be used as low as −70 °C, which meets the requirements for use in an ultra-low temperature environment.

The moisture stability of the HAG_x_ hydrogels was studied by detecting the weight loss. In Figure 3b, the tests are carried out at 25 °C for 15 days, and Figure S5 shows the photos of HAPAA and HAG_x_ hydrogels before and after 15 days of storage. The inset of Figure 3b shows the weight changes of HAG_x_ hydrogels within 24 h. The weight retention rate of the HAPAA hydrogel is only 83% after 24 h, and that of the HAG_13_ hydrogel is almost 100%, in contrast. After 15 days, the weights of the tested hydrogels are basically constant. However, the weight retention rate of HAPAA is only 32%. From the photos in Figure S5, we can observe that HAPAA is almost completely dehydrated with severe volume shrinkage, as it turns into a dense and hard, dry gel. As expected, the appearance change of the HAG_x_ hydrogels is much smaller in comparison. Moreover, with the increase of glycerol, the appearance change is less obvious, which means water evaporation gradually decreases. In particular, the weight retention rate of HAG_13_ hydrogel is up to 93% when the mass ratio of water–glycerol is 1:3, showing excellent moisture stability. In practical applications, it has great significance because it is fundamental to stable performance in mechanical and electrical properties during the long-term use of the hydrogels.

As is known, the interaction strength of hydrogen bonds in the water–glycerol mixed solution is much stronger in water solutions. Besides, Lu [36] confirmed that the water–glycerol mixture has strong interactions with PAA molecular chains by hydrogen bonding. The strong interaction of these hydrogen bonds can effectively prevent ice formation at subzero temperatures and weaken the water evaporation at room temperature or at a higher temperature. This is also the main reason why our hydrogel has excellent moisturizing properties.

### 3.2. Adhesive Property and Transparency

For signal detection, most flexible electronics are required to be attached on different surfaces with the help of some extra adhesives or bandages due to their lack of stickiness. Fortunately, the HAG_x_ hydrogels prepared in this study are born with good stickiness and can be directly adhered to different substrates. Figure 3c quantitatively displays the adhesive strength between HAG_11_ hydrogels and different substrates, including stainless steel, polyester, cardboard, and copper plates, and the corresponding adhesive strengths are 260.45 ± 11.5 kPa, 30.5 ± 1.4 kPa, 222.0 ± 6.4 kPa, and 55.3 ± 14.6 kPa, respectively. The adhesive strengths in Figure 3c are much higher than most of the reported adhesive hydrogels. The inset shows that two stainless steel plates bonded with HAG_11_ hydrogel can lift a weight of 1 kg firmly, specifically demonstrating exceptional adhesiveness.

On the one hand, the HAG_x_ hydrogels can form strong hydrogen bonds with a metal oxide layer on metal materials; the −COO^−^ in the HAG_x_ hydrogels facilitates the formation of metal complexation interactions [50,51]. On the other hand, glycerol has an advantage over water in viscosity [52,53], making the interaction with different substrates tighter. In addition, as confirmed by Lu et al. [36], compared with water, glycerol has stronger interactions with both the HAPAA polymer network and substrates, which greatly improves the adhesive strength.

For the application of the sensors on the skin surface, the requirements of visual and aesthetic are of great concern. In the case of HAG_x_ hydrogels, there is no addition of the other colored conductive fillers. In visible spectra, the HAG_x_ hydrogels show excellent transparency (Figure 3d). At 550 nm, the transmittance of the HAPAA hydrogel and the HAG_x_ hydrogel is 70.3% and 92.8%, respectively. The inset of Figure 3d has three photos, which are taken by mobile phone. The difference is that (i) is taken directly without obstruction, and the camera is covered with a piece of 2 mm HAG_11_ hydrogel and HAPAA hydrogel in (ii) and (iii)**,** respectively. Comparing (ii) and (iii), (ii) was clearer than (iii), which also confirmed that the HAG_11_ hydrogel has better transparency.

### 3.3. Ultra-Stretchable Properties

**Figure 4:**
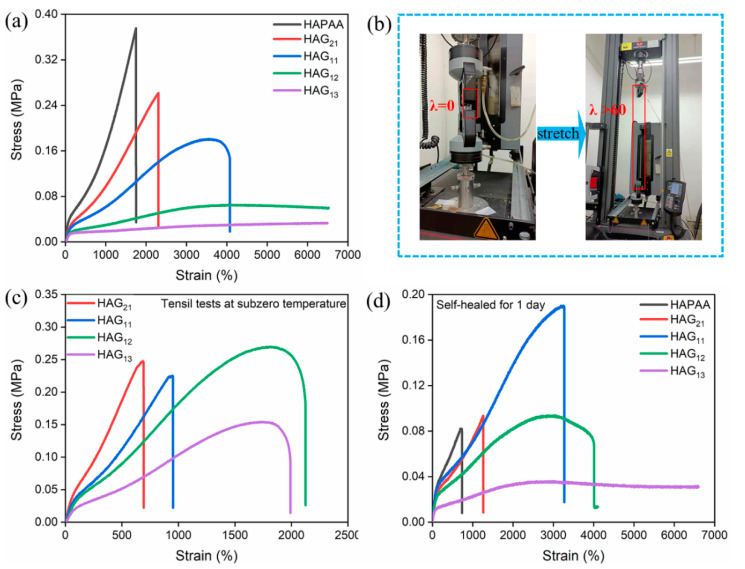
(**a**) Stress–strain curves of HAPAA and HAG_x_ hydrogels. (**b**) The HAG_12_ hydrogel can be stretched to more than 6000%. (**c**) The stress–strain curves of HAPAA and HAG_x_ hydrogels after 24 h storage at −20 °C. The tests were done immediately after the hydrogels were taken out. (**d**) The stress–strain curves of the healed HAPAA and HAG_x_ hydrogels after 24 h at room temperature.

Figure 4a shows the stress–strain curves of HAG_x_ hydrogels at room temperature (25 °C). By tuning the mass ratio of glycerol, the mechanical strength and stretchability can be adjusted over a wide range. In general, with the increase of glycerol, the tensile strength and modulus of HAG_x_ hydrogels decrease (Figure S6a,b), while the stretchability increases gradually. Of particular note, is that when the water–glycerol mass ratios reach 1:2 and 1:3, the HAG_12_ and HAG_13_ hydrogels show excellent stretchability, which can be further stretched to over 6000% without breaking, due to the limitation of the test equipment (Figure 4b, Video S1). It is worth noting that, under 60 times the stretch of the original length, the cross-sectional area of the specimens falls severely. Thus the true stress is actually much larger than the tested values. For the HAG_11_ hydrogel, the stretchability is more than 4000%, and the tensile strength is 0.17 MPa. 

The addition of glycerol significantly affects the mechanical properties of HAPAA hydrogel. This can be explained by the following three points: (1) the polarity of glycerol is lower than that of pure water, and the interaction strength of hydrophobic associations is greatly affected by the polarity of the solution [44,54]. Compared with the aqueous solution, the mixed water–glycerol solution is less polar, and the micelles associate loosely. So, the association strength is weaker, and the chain segments can move easily, increasing stretchability; (2) glycerol contains a large number of hydroxyl groups, which form much more intermolecular hydrogen bonds with water and HAPAA. The strong van der Waals force has a significant influence on the mechanical properties of hydrogels; (3) for most of the hydrogels, there is only one kind of dynamic crosslinking. In this study, a double association system of hydrophobic association and hydrogen bonds leads to great stretchability.

In addition to the room temperature, the mechanical tests at subzero temperatures were also taken. The specimens used for tests were stored at −20 °C for 48 h and then taken out quickly before testing. Figure 4c shows that the anti-freezing HAG_x_ hydrogels still have great elasticity and flexibility under subzero temperatures. In contrast, the HAPAA hydrogel loses its mechanical performance under −20 °C. Compared with the tests at room temperature, the tensile strength and modulus of the HAG_x_ hydrogels all show an improvement (Figure S6c). One reasonable explanation is that under subzero temperatures, the movement of molecular chain segments slows down, and the presence of glycerol introduces many hydrogen bonds [33,36]. By comparison, we also found that with the increase of glycerol, the stretchability and strength of the HAG_x_ hydrogels increase first and then decrease. When the mass ratio of water to glycerol is 1:2, the HAG_12_ hydrogel exhibits good stretchability and mechanical strength with 2125% and 0.27 MPa, respectively. However, a large amount of glycerol destroys the microdomains of hydrophobic association. Thus, the mechanical performance has a decline when the mass ratio of water–glycerol is 1:3.

### 3.4. Self-Healing Property

The self-healing properties of the HAPAA and HAG hydrogels were investigated in both mechanical and electric performances. The mechanical properties of hydrogels after self-healing were tested by the tensile machine. The tested hydrogels were cut through the middle by a scalpel and brought into contact immediately. Figure 4d is the stress–strain curve of HAPAA and HAG hydrogels after self-healing for 24 h at room temperature. The self-healing efficiency of HAG_11_ reaches 89.4%. In particular, the self-healed HAG_13_ hydrogel cannot even be broken when the strain exceeds 6000% (similarly to the original one), and the self-healing efficiency of HAG_12_ and HAG_13_ is 100%. One possible explanation is that the hydrogels lost some water during the healing process. The reduction of water strengthens the association of hydrogen bonds in the hydrogel [39]. By comparison, the healing efficiency of HAPAA and HAG_21_ hydrogels is only 29.7% and 35.8%, respectively. 

In view of the practical application, the HAG_11_ hydrogel has a balanced performance both in strength and stretchability, which is much better than most reported stretchable anti-freezing hydrogels (Table S2). In addition, the HAG_11_ hydrogel also shows excellent tensile performance in subzero temperatures and good self-healing capabilities. Therefore, the HAG_11_ hydrogel was chosen to perform the following tests unless otherwise stated.

Figure S7 vividly illustrates the self-healing behavior of the electrical properties. The HAG hydrogel was used as a conductor in the circuit to light up a small light bulb at a voltage of 5 V. Figure S7a–c are the original sample, the reconnected hydrogel after cutting it off, and after self-healing for 6 h at room temperature, respectively. In Figure S7b, we can observe that after the immediate reconnection, the light was lit up but showed a lower brightness. However, after healing for 6 h, the light is almost as bright as before. This result proves that the electrical performance of the HAG hydrogel was fully recovered after healing.

The self-healing ability of the HAG hydrogel at subzero temperatures was also investigated. Figure 2c illustrates the surface microscope images of the HAG hydrogel before the cut, after the cut, and after healing (−20 °C for 3 d), respectively. After healing, the conspicuous scar on the gel surface was essentially healed except for a vague scar. Figure 2d clearly shows that the HAG hydrogel can be stretched to about seven times its original length after self-healing for 3 days at −20 °C. These results showed that HAG hydrogels have remarkable self-healing ability at subzero temperatures.

The excellent self-healing ability of HAG hydrogels can be explained by the following aspects: (1) the addition of glycerol weakens the hydrophobic association, loosens the interaction strength of the micelles, and increases their mobility. When the hydrogels are damaged, the micelles can reorganize quickly, facilitating the reconnection of the damaged sites; (2) glycerol contains many hydroxyl groups, which promotes the formation of intermolecular hydrogen bonds with water, glycerol, and HAPAA. A hierarchical non-covalent crosslinking of hydrophobic association and hydrogen bonds bring about remarkable stretchability and damages self-healing [55].

### 3.5. Strain Sensors

The HAG hydrogel is an economical material when applied for wearable strain sensors. Due to the addition of CTAB charged micelles and APS in the binary water–glycerol solution, the HAG_11_ hydrogel can be used as a strain sensor even without conductive fillers and conductive polymers. The free-moving ions in HAG hydrogels can be ionized in water instead of glycerol, which helps form molecular-level ion-conducting channels in the HAG hydrogels [35,55]; the 3D network structure of HAG hydrogels provides the free-moving ions mobile channel [56,57]. In addition, the charged micelles make the micelle-bridging effect possible.

**Figure 5:**
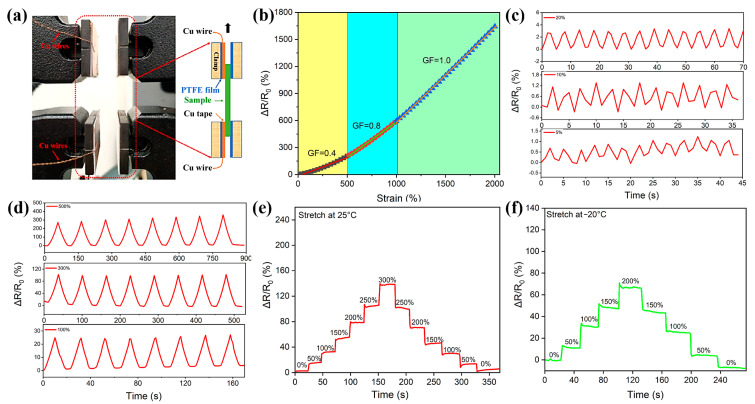
(**a**) The test method for the strain sensor based on the HAG_11_ hydrogel. (**b**) The relative resistance changes of the HAG_11_ hydrogel. (**c**,**d**) The relative resistance changes from small strains to big strains. (**e**,**f**) The relative resistance changes upon different strains at room temperature and −20 °C, respectively.

As a demonstration, some tests were designed to assess the feasibility of HAG hydrogels as strain sensors. Strain sensitivity is evaluated through the gauge factor (GF = (R − R_0_)/εR_0_; R_0_ and R represent the original resistance without deformation and the resistance at a certain strain ε, respectively) (Figure 5a,b). The GF can be determined as 0.4 from the slope of the fitting curve within stage A (0% < ε < 500%), and then increased to 0.8 (stage B, 500% < ε < 1000%) and 1.0 (stage B, 1000% < ε < 2000%). To further evaluate the strain sensitivity of the HAG hydrogel, a series of cyclic tensile tests with different strains were conducted. Figure 5c,d are the resistance variations under relatively small and large strains, respectively. They show that a regular resistance response can be detected at strains of 5%, 10%, and 20%. Also, they are sensitive to large strains (100%, 300%, and 500%). In addition, as shown in Figure 5e, the resistance variation presents a prominent step-wise growth when the strain increases from 0% to 300% by a step of 50% at room temperature. Subsequently, with the stress releasing, the hydrogel restores to its original shape, and the recovered resistance is almost consistent with the value before the changes. Note that the hydrogel also shows similar changes in resistance (Figure 5f) at −20 °C, showing its low temperature sensing capability. The above results indicate that HAG hydrogels sensors have good stability and durability in electromechanical properties.

## 4. **Conclusions**

A conductive, anti-freezing, self-healing, stretchable, and adhesive HAG_x_ hydrogel based on hydrophobic associated polyacrylic acid (HAPAA) was successfully synthesized and prepared by replacing the water with a binary water–glycerol solution. These HAG_x_ hydrogels not only had an excellent anti-freezing property at a range of −70 to 25 °C, but also showed a good moisturizing ability. The weight retention rate was even as high as 93% after 15 days of natural storage; however, the weight retention rate of the ordinary HAPAA hydrogel was only 32%, in contrast. Our HAG_x_ hydrogels also had a superior stretchable and self-healing ability. They could be stretched to more than 6000% without any breaking and had a 100% self-healing efficiency after being cut in half and reconnected for 24 h. Surprisingly, the HAG_x_ hydrogels even had a good self-healing ability at subzero temperatures. 

HAG_x_ hydrogels in this study also had eye-catching adhesive properties and transparency, and the dodecyltrimethylammonium bromide (CTAB) cationic micelles and initiator ions (APS) endowed these hydrogels with electrical conductivity. The as-prepared HAG_x_ hydrogel was sealed as a stretch sensor. The tests confirmed that a stretch sensor based on HAG_x_ hydrogels not only had good response at a strain of 5%, 10%, and 20%, but also showed high sensitivity to large strains (100%, 300%, and 500%). Moreover, the stability and durability of HAG_x_ hydrogels sensors were also good.

## Data Availability

The data presented in this study are available on request from the corresponding author.

## Supplementary Materials

The following are available online at https://www.mdpi.com/article/10.3390/ma14206165/s1, Figure S1: ATR-FTIR spectra of hydrogels, Figure S2: DFT-optimized structure of glycerol, H_2_O, and HAPAA, Figure S3: storage modulus and the loss modulus of hydrogels, Figure S4: relationship between the freezing points and the glycerol mass ratio in mixed solutions, Figure S5: comparison of the freshly prepared hydrogels and hydrogels after a storage time of 15 days, Figure S6: tensile stress and modulus of hydrogels, Figure S7: circuits comprised of the hydrogel and a green LED light, Table S1: compositions of the HAPAA hydrogel and HAG_x_ hydrogels, Table S2: comparison of the HAG_11_ hydrogel with other antifreeze stretchable hydrogels in mechanical strength, Table S3: Interaction energies in H_2_O-HAPAA, glycerol-HAPAA, and glycerol-H_2_O-HAPAA by DFT calculations, Video S1: real-time ultra-stretching experiment of the hydrogel.
