# Supplementary material for: Ultra-Stretchable and Self-Healing Anti-Freezing Strain Sensors Based on Hydrophobic Associated Polyacrylic Acid Hydrogels
Source: Materials (Basel). 2021 Oct 18;14(20):6165. doi: 10.3390/ma14206165 (PMC8538095; doi:10.3390/ma14206165)
Supplement: Supplementary file 1 [file materials-14-06165-s001.zip › Supporting Information.pdf]

Supporting Information

Ultra-Stretchable and Self-Healing Anti-Freezing Strain Sensors based on Hydrophobic Associated Polyacrylic Acid Hydrogels

Shuya Yin,[a] Gehong Su,[a,b] Jiajun Chen,[a] Xiaoyan Peng,[a] and Tao Zhou [a,]*

[a] State Key Laboratory of Polymer Materials Engineering of China, Polymer Research Institute, Sichuan University, Chengdu 610065, China; [b] College of Science, Sichuan Agricultural University, Ya'an, 625014, China

*Corresponding author. Tel.: +86-28-85402601; Fax: +86-28-85402465; E-mail address: zhoutaopoly@scu.edu.cn (T. Zhou)

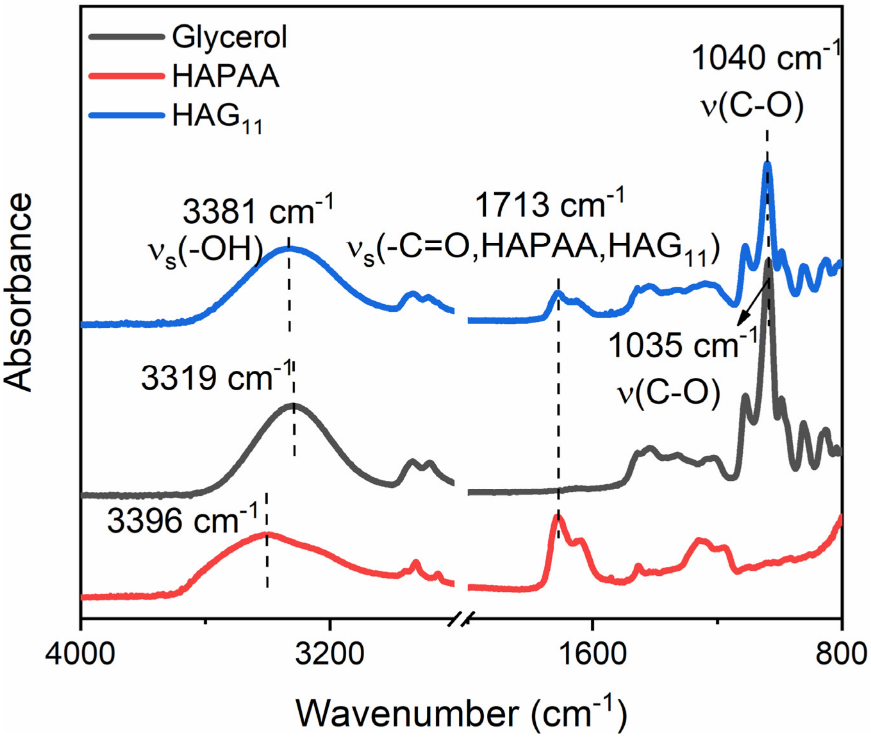

Figure S1. ATR-FTIR spectra of glycerol, HAPAA, and HAG_{11} hydrogels.

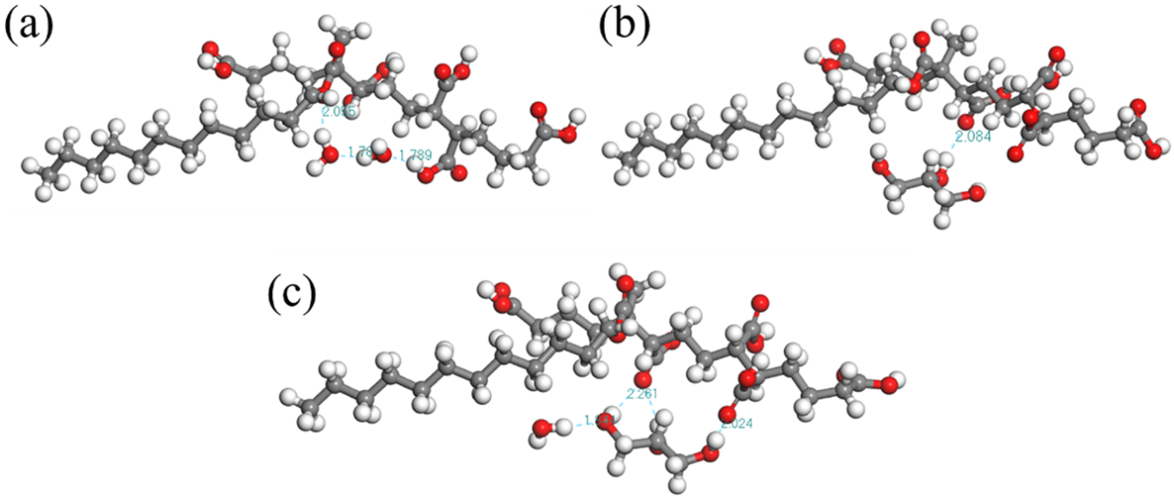

Figure S2. DFT-optimized structure of glycerol, H_2O, and HAPAA. The interaction models of **(a)** H_2O-HAPAA, **(b)** glycerol-HAPAA, and **(c)** H_2O-glycerol-HAPAA.

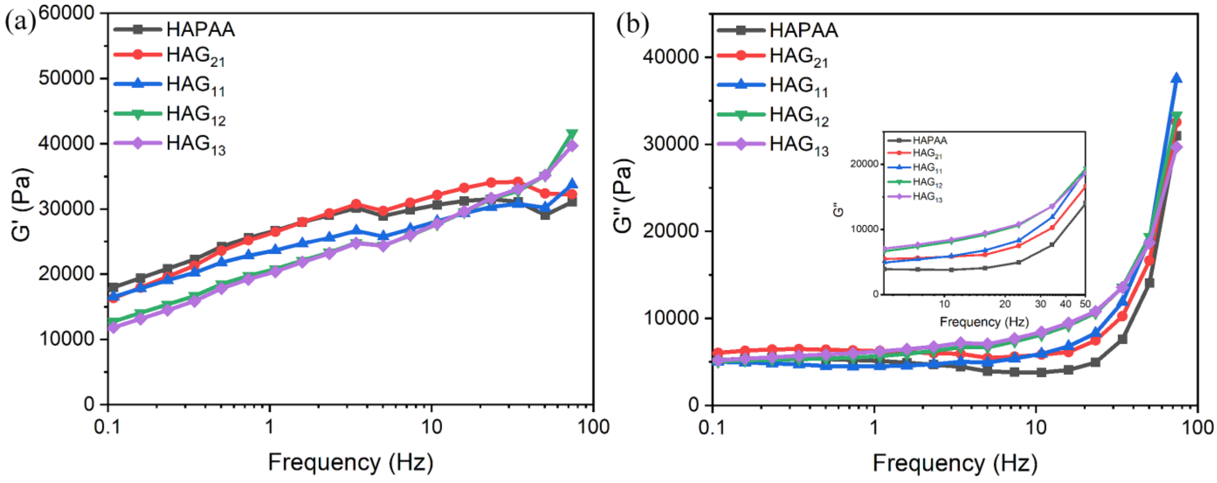

Figure S3. (a) Storage modulus (G'), and **(b)** the loss modulus (G'') of the HAPAA and HAG_x hydrogels.

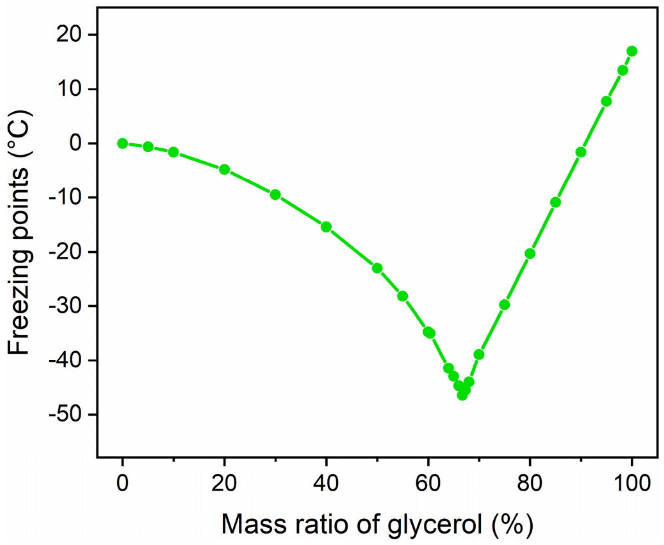

Figure S4. Relationship between the freezing points and the glycerol mass ratio in the glycerol-water mixed solutions.[1]

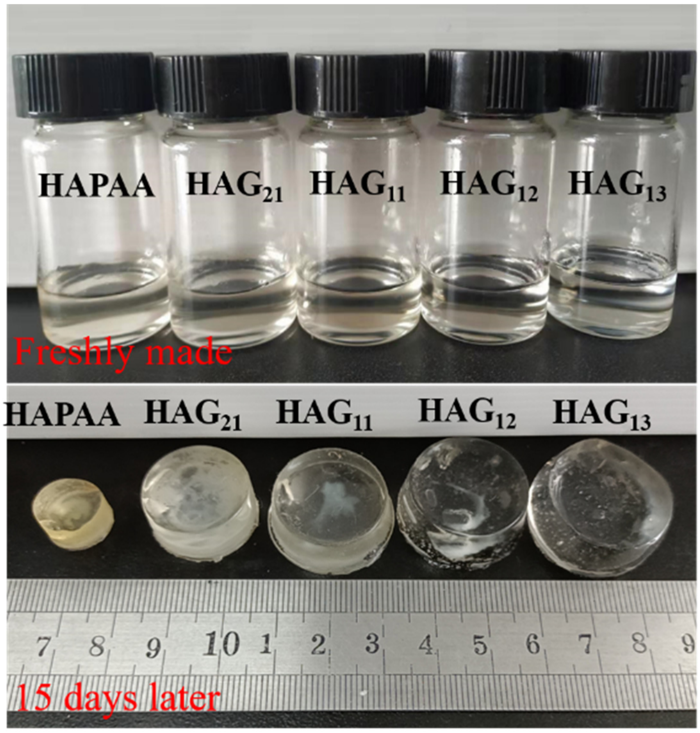

Figure S5. Comparison of the freshly prepared HAPAA and HAGx hydrogels (top) and after a storage time of 15 days (bottom).

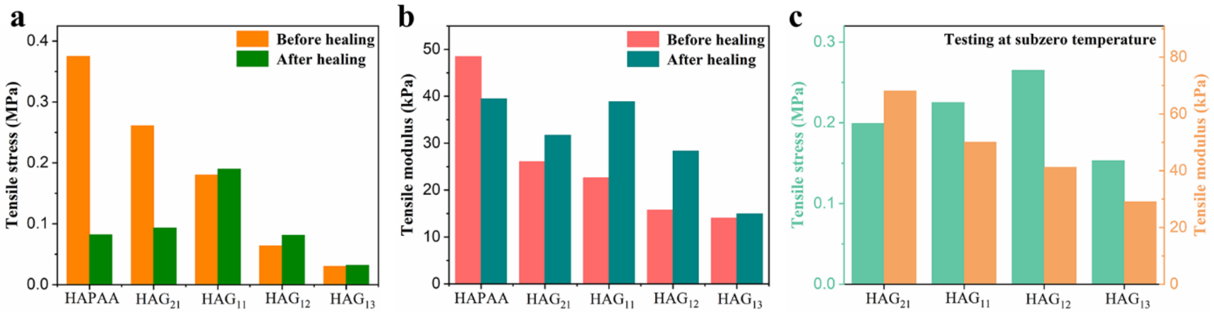

Figure S6. **(a-b)** The tensile stress and (b) modulus of HAPAA and HAG$_x$ hydrogels before and after self-healing (RT, 24h). **(c)** The tensile stress and modulus of HAPAA and HAG$_x$ hydrogels at subzero temperature.

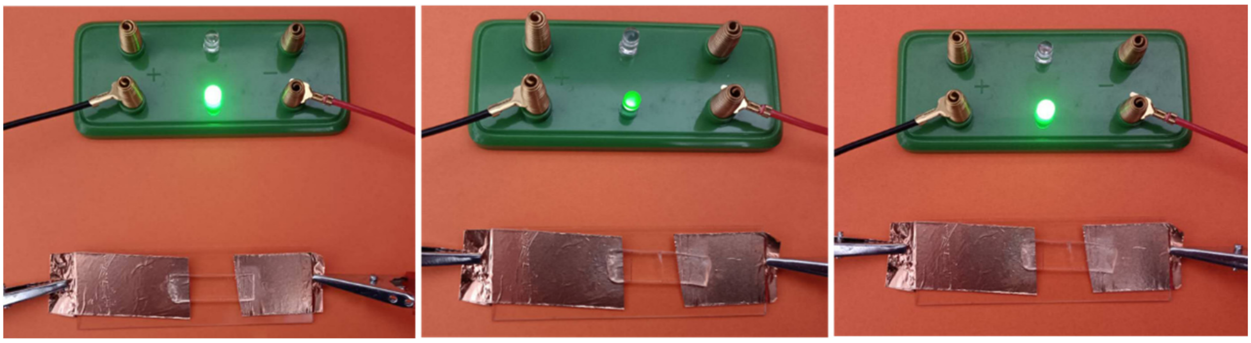

Figure S7. Circuits comprised of the HAG$_{11}$ hydrogel and a green LED light: **(a)** original, **(b)** cutting off and being contacted immediately, **(c)** self-healing for 6 h.

Table S1. Compositions of the HAPAA hydrogel and HAG_x hydrogels

Samples	AA(g)	LMA(g)	CTAB(g)	H_2O(g)	Glycerol(g)	APS(mg)	H_2O/Glycerol (wt/wt)
HAPAA	2.5	0.2	0.3	7.00	0	1	--
HAG_{21}	2.5	0.2	0.3	4.67	2.33	1	2:1
HAG_{11}	2.5	0.2	0.3	3.50	3.50	1	1:1
HAG_{12}	2.5	0.2	0.3	2.33	4.67	1	1:2
HAG_{13}	2.5	0.2	0.3	1.75	5.25	1	1:3

Table S2. The comparison of the HAG_{11} hydrogel with other antifreeze stretchable hydrogels in mechanical strength

Materials	Antifreeze temperature (℃)	Stretchability (%)	Tensile Strength (MPa)	Self-healing	Refs.
PVA (EG)	-40	1000	1.5	Yes (freeze thawing)	2
PAMPS/PAAm (EG/LiCl)	-80	225	0.57	No	3
PAAm ($CaCl_2$)	-54	450	0.12	No	4
PAAm/PVA (EG)	-40	--	--	No	5
PAAm/casein (LiCl)	-20	1450	0.16	No	6
PVA-TA@talc (EG)	-30	700	0.6	No	7
PVA-PAA (EG)	-25	550	0.025	No	8
PAA-PANI (Glycerol)	-26	1000	0.035	Yes	9
PAA-PAAm (Glycerol)	-20	600	0.075	No	10
HAG_{11} (Glycerol)	-70	4000	0.18	Yes	This work

Table S3. Interaction energies in H_2O-HAPAA, glycerol-HAPAA, and glycerol-H_2O-HAPAA by DFT calculations.

Interaction pair	Interaction energy (Ha)
H_2O-HAPAA	-0.02013
Glycerol-HAPAA	-0.00994
Glycerol-H_2O-HAPAA	-0.02651

References

1. Lane, L. B., Freezing Points of Glycerol and Its Aqueous Solutions. *Industrial & Engineering Chemistry* **1925**, *17*, 924-924.
2. Rong, Q.; Lei, W.; Chen, L.; Yin, Y.; Zhou, J.; Liu, M., Anti-Freezing, Conductive Self-Healing Organohydrogels with Stable Strain-Sensitivity at Subzero Temperatures. *Angew Chem. Int. Ed. Engl.* **2017**, *56*, 14159-14163.
3. Lou, D.; Wang, C.; He, Z.; Sun, X.; Luo, J.; Li, J., Robust Organohydrogel with Flexibility and Conductivity across the Freezing and Boiling Temperatures of Water. *Chem. Commun.* **2019**, *55*, 8422-8425.
4. Morelle, X. P.; Illeperuma, W. R.; Tian, K.; Bai, R.; Suo, Z.; Vlassak, J. J., Highly Stretchable and Tough Hydrogels Below Water Freezing Temperature. *Adv. Mater.* **2018**, *30*, 1801541.
5. Liao, H.; Guo, X.; Wan, P.; Yu, G., Conductive Mxene Nanocomposite Organohydrogel for Flexible, Healable, Low-Temperature Tolerant Strain Sensors. *Adv. Funct. Mater.* **2019**, *29*. 1904507.
6. Wu, X.; Liao, H.; Ma, D.; Chao, M.; Wang, Y.; Jia, X.; Wan, P.; Zhang, L., A Wearable, Self-Adhesive, Long-Lastingly Moist and Healable Epidermal Sensor Assembled from Conductive Mxene Nanocomposites. *J. Mater. Chem. C* **2020**, *8*, 1788-1795.
7. Guan, L.; Yan, S.; Liu, X.; Li, X.; Gao, G., Wearable Strain Sensors Based on Casein-Driven Tough, Adhesive and Anti-Freezing Hydrogels for Monitoring Human-Motion. *J. Mater. Chem. B* **2019**, *7*, 5230-5236.
8. Ge, G.; Yuan, W.; Zhao, W.; Lu, Y.; Zhang, Y.; Wang, W.; Chen, P.; Huang, W.; Si, W.; Dong, X., Highly Stretchable and Autonomously Healable Epidermal Sensor Based on Multi-Functional Hydrogel Frameworks. *J. Mater. Chem. A* **2019**, *7*, 5949-5956.
9. Ge, G.; Lu, Y.; Qu, X.; Zhao, W.; Ren, Y.; Wang, W.; Wang, Q.; Huang, W.; Dong, X., Muscle-Inspired Self-Healing Hydrogels for Strain and Temperature Sensor. *ACS Nano* **2020**, *14*, 218-228.
10. Han, L.; Liu, K.; Wang, M.; Wang, K.; Fang, L.; Chen, H.; Zhou, J.; Lu, X., Mussel-Inspired Adhesive and Conductive Hydrogel with Long-Lasting Moisture and Extreme Temperature Tolerance. *Adv. Funct. Mater.* **2018**, *28,* 1704195.
